# Helicase and Its Interacting Factors: Regulation Mechanism, Characterization, Structure, and Application for Drug Design

**DOI:** 10.1155/2015/909047

**Published:** 2015-01-28

**Authors:** Cheng-Yang Huang, Yoshito Abe, Huangen Ding, I-Fang Chung

**Affiliations:** ^1^School of Biomedical Sciences, Chung Shan Medical University, Taichung, Taiwan; ^2^Department of Medical Research, Chung Shan Medical University Hospital, Taichung, Taiwan; ^3^Department of Protein Structure, Function and Design, Kyushu University, Fukuoka, Japan; ^4^Department of Biological Sciences, Louisiana State University, Baton Rouge, LA, USA; ^5^Institute of Biomedical Informatics, National Yang-Ming University, Taipei, Taiwan

Helicases are motor proteins that separate nucleic acid duplexes and/or displace protein in reactions fueled by the binding and hydrolysis of nucleoside triphosphate (NTP). Because of their essential roles in all aspects of nucleic acid metabolism, helicases encoded by bacteria, viruses, and human cells are widely studied targets for new antiviral, antibiotic, and anticancer drugs. Recent evidence indicates that some accessory proteins can regulate their helicase and/or translocase activities. Knowledge of structure-activity relationships has led to the development of successful therapies, regulation modes, new DNA/protein interacting models, and novel inhibitors to deeply understand the acting mechanism of helicases and/or their interacting factor.

In this special issue, we presented original research papers and reviews on the topics of how DEAH/RHA helicases can be regulated by G-patch proteins (J. Robert-Paganin et al.), a possible role of the Mcm2-7 replicative helicase as a promising chemotherapeutic target for anticancer drug development (N. E. Simon and A. Schwacha), crystal structural analyses of* Deinococcus radiodurans* RecQ helicase (S.-C. Chen et al.) and a conserved hypothetical protein MJ0927 from* Methanocaldococcus jannaschii* (S.-C. Chen et al.), identification and characterization of human DNA helicase Rtel1 possessing a redox active iron-sulfur cluster (A. P. Landry and H. Ding), a role of the C-terminal domain of SSB in determination of the ssDNA binding site size (Y.-H. Huang and C.-Y. Huang), and the structural insight into the DNA-binding mode of the primosomal proteins PriA, PriB, and DnaT (Y.-H. Huang and C.-Y. Huang). We hope that the readers will find in this special issue accurate data, significant results, and updated reviews.

*Cheng-Yang Huang* *Yoshito Abe* *Huangen Ding* *I-Fang Chung*

